# Corrosion Resistance of AISI 442 and AISI 446 Ferritic Stainless Steels as a Support for PEMWE Bipolar Plates

**DOI:** 10.3390/ma16041501

**Published:** 2023-02-10

**Authors:** Mircea Laurentiu Dan, Andrea Kellenberger, Delia Duca, Nicolae Vaszilcsin, Corneliu Marius Craciunescu, Ion Mitelea, Aurel Ercuta, Sigrid Lædre, Thulile Khoza

**Affiliations:** 1 Faculty of Industrial Chemistry and Environmental Engineering, Politehnica University Timisoara, Piata Victoriei 2, 300006 Timisoara, Romania; 2 Faculty of Mechanical Engineering, Politehnica University Timisoara, Piata Victoriei 2, 300006 Timisoara, Romania; 3 SINTEF Industry, 7465 Trondheim, Norway

**Keywords:** PEMWE, water electrolysis, bipolar plate, ferritic stainless steel, corrosion resistance

## Abstract

Cost reduction in bipolar plates in proton exchange membrane water electrolyzers has previously been attempted by substituting bulk titanium with austenitic stainless steels protected with highly conductive and corrosion-resistant coatings. However, austenitic steels are more expensive than ferritic steels due to their high nickel content. Herein we report on the corrosion resistance of two high chromium ferritic stainless steels, AISI 442 and AISI 446, as an alternative material to manufacture bipolar plates. Electrochemical corrosion tests have shown that AISI 442 and AISI 446 have similar corrosion resistance, while AISI 446 reveals more noble corrosion potential and performs better during potentiostatic stress tests. The current density obtained during polarization at 2 V versus the standard hydrogen electrode (SHE) is 3.3 mA cm^−2^, which is more than two times lower than on AISI 442. Additionally, surface morphology characterization demonstrates that in contrast to AISI 442, AISI 446 is not sensitive to intercrystalline or pitting corrosion. Moreover, EDX energy dispersion analysis of AISI 446 reveals no differences in the chemical composition of the surface layer compared to the base material, as a confirmation of its high corrosion resistance. The results of this work open up the perspective of replacing austenitic stainless steels with less expensive ferritic stainless steels for the production of components such as bipolar plates in proton exchange membrane water electrolyzers.

## 1. Introduction

In recent decades, climate change has become evident, and decision-makers around the world have understood that significant investments need to target the renewable energy sector in order to curb the further increase in carbon dioxide content in the atmosphere. Production of electricity from intermittent energy sources such as solar and wind is increasing, but the discontinuous operation of such production facilities does not align well with the demand. In order to utilize all the energy produced, hydrogen is foreseen to play an important role as an energy carrier [1]. Water electrolysis is used for hydrogen production. However, for economic reasons, the share of electrolytic hydrogen in world production does not exceed 5% [2]. Proton exchange membrane water electrolyzers (PEMWEs) have several advantages over alkaline water electrolyzers, such as high energy efficiency, high current density operation and pressure. Moreover, PEMWEs can be operated under dynamic conditions with fluctuating power loads, making them suitable for grid balancing where renewables are used [3,4,5,6,7].

One of the most important components of PEMWE stacks is the bipolar plates (BPPs), which isolate individual cells in a stack and conduct electrical current in addition to facilitating thermal management within the stack. BPPs are the main cost drivers, accounting for more than 50% of the total stack cost [8]. The high cost of this component is mainly associated with the utilization of expensive Ti materials [9] to withstand acidic and oxidizing conditions at the anode side of the electrolyzer and complex machining labor during manufacturing. Frequently, a thin Pt coating is applied on top of the BPPs to prevent surface anodizing of titanium [10], which would lead to increased interfacial contact resistance (ICR) during operation. The role of the precious metal coating is to maintain the ICR between BPPs and the gas diffusion layer (GDL) or porous transport layer (PTL) at very low values. Lædre et al. [11] studied the corrosion behavior and ICR evolution of several metals and alloys under simulated PEMWE conditions: Ti (grade 2), Ta, Nb, W, Mo, AISI 316L, AISI 304L, 254 SMO and Inconel 625. Weight loss, potentiostatic and potentiodynamic polarization measurements in 0.1 M Na_2_SO_4_ solution acidified to pH = 5.5 with H_2_SO_4_ indicated the highest corrosion resistance for Ti, Ta and Nb. However, both Ta and Nb showed a significant increase in ICR after polarization, attributed to the passive oxide layer formed on the metal surface. The use of stainless steel (SS) [10,12] or copper [13] coated with highly corrosion-resistant layers of dense Ti, Ti/Nb and Nb has also been reported as a possible alternative to replace titanium for the manufacture of BPPs.

There is currently no set target for the corrosion resistance and ICR of materials used in the production of BPPs for PEMWEs. The US Department of Energy (DoE) has set a number of targets for bipolar plates in PEM fuel cells (PEMFCs) [14], including a maximum corrosion current density of 1 μA cm^−2^ and a maximum contact resistance of 10 mΩ cm^2^ between the GDL and the BPP [14]. In situ ICR measurements in PEMFC during operation showed ICR values close to 5 mΩ cm^2^ for Au-coated stainless steel BPPs [15]. However, BPPs in PEMWEs face much more aggressive conditions than in PEMFCs, especially on the anodic side (acid medium, the presence of dissolved oxygen and advanced anodic polarization) [16]. This is why the corrosion resistance testing protocols of BPPs in PEMWE also include potentiostatic stress test (PST) at potentials up to 2 V [12], although a recent study questions this limit as not representative of the local potential of BPP during real operating conditions [17]. Moreover, high corrosion resistance is also important to avoid the release of metal cations, which migrate into the membrane and leads to performance loss by blocking the active sites and increasing the electrical resistance [18,19]. It should be emphasized that even minor imperfections in the structure of the protective layers, induced either during production or during use, can expose the base metal, which is why the metal support must be corrosion-resistant.

Austenitic steels are expensive due to their high nickel content, and our research has thus focused on the high chromium ferritic stainless steels used as a support for BPPs. For PEMFCs, there are numerous reviews on the current research of materials for BPPs [20,21,22,23]. Thus, several studies reported the use of both austenitic [24,25,26,27,28,29] and ferritic stainless steels [30,31,32,33,34] as a support material for BPPs, with or without protective films, but in PEMFCs they operate in less aggressive conditions than in PEMWEs. The corrosion resistance of austenitic steels can be additionally improved by laser heat treatments [35,36]. Ferritic stainless steels have a high resistance to stress-corrosion-cracking (SCC) in chlorinated environments and in strongly oxidizing environments, such as nitric acid. They also show resistance to oxidation at high temperatures and no tendency to pitting and crevice corrosion in chlorinated environments [37]. In addition, molybdenum alloying further improves local corrosion resistance by forming a passive MoO_2_ layer in the active region of stainless steel [38], while silicon and aluminum increase resistance to oxidation at high temperatures. The disadvantage of these steels is that the higher the chromium content, the greater the sensitivity to hardening by intergranular precipitation of chromium carbides and nitrides, and the tenacity and resistance to intercrystalline corrosion will decrease [37].

This paper presents the results obtained in the study of the anticorrosive properties of ferritic stainless steels AISI 442 and 446. The corrosion resistance was evaluated by potentiodynamic and potentiostatic polarization, and the results were confirmed by electrochemical impedance spectroscopy. Considering the fact that BPPs represent the “backbone” of PEMWEs, the mechanical properties of AISI 442 and 446 steels were determined: tensile strength (*R*_m_), yield strength (*R*_p0.2_), elongation (A) and Brinell hardness (HB). In addition, the structure and morphology of the stainless steel samples subjected to the potentiostatic stress test were investigated by scanning electron microscopy. The obtained results indicate good corrosion resistance and open up the perspective of using less expensive ferritic stainless steels for the production of components such as bipolar plates in proton exchange membrane water electrolyzers.

## 2. Materials and Methods

The anticorrosive properties of the AISI 442 and 446 stainless steels were determined in 0.1 mol L^–1^ Na_2_SO_4_ solution, acidified with H_2_SO_4_ to pH = 5. In order to simulate the working environment of the anode side of PEMWEs as accurately as possible, the test solutions were saturated with O_2_ (purity ≥ 99.95%), and NaF was added so that F^–^ concentration reached 0.1 ppm. The tested samples were made of AISI 442 and AISI 446 stainless steel sheets (thickness 1.8 mm), included in the specifications of ASTM A176-74 (Chromium stainless flat products), A 511 (Seamless stainless steel mechanical tubing), A268-74 (Ferritic stainless steel tubing for general service) and also in ASME code and AISI and SAE specifications. Table 1 gives the chemical composition of the tested and standard AISI 442 and 446 stainless steels.

Anhydrous sodium sulfate (Riedel-de Haën, puriss. p.a. min. 99%), sulfuric acid (Honeywell Fluka, puriss. p.a. 96–97%) and sodium fluoride (Honeywell Fluka, puriss. p.a. min. 99%) were used to prepare the solutions.

All electrochemical measurements were performed in a conventional corrosion cell equipped with three electrodes: a working electrode (AISI 442 or 446), a counter electrode (Pt mesh) and a reference electrode (Ag/AgCl, sat. KCl) placed in the cell via a bridge. All potential values were measured against this reference electrode (*E*_Ag/AgCl_ = +0.197 V) and afterward converted versus the standard hydrogen electrode (SHE). A BioLogic SP 150 potentiostat (BioLogic Science Instruments, Grenoble, France) served to conduct open circuit potential, potentiodynamic and potentiostatic polarization, as well as electrochemical impedance measurements.

Firstly, the open circuit potential (OCP) was measured for 1 h for each sample. Then, potentiodynamic polarization curves were obtained, and the Tafel extrapolation method was used to determine corrosion current density *i_corr_* and corrosion potential *E_corr_*, together with the anodic and cathodic Tafel slopes *b_a_* and *b_c_*, respectively. The polarization resistance *R_p_* resulted from the Stern–Geary relation (Equation (1)) [39] and the determined corrosion parameters.

(1) $$R_{p}=\frac{b_{a}\times b_{c}}{i_{corr}\times 2.303\left(b_{a}+b_{c}\right)}$$

Electrochemical impedance spectroscopy (EIS) was used to confirm the results obtained by the Tafel extrapolation method. The electrochemical impedance spectra were plotted at the corrosion potential, in the frequency range of 100 kHz–10 mHz, using a sinusoidal signal of 5 mV amplitude.

Further information on the stability of AISI 442 and 446 steel resulted from the potentiostatic stress tests. As materials used for BPPs manufacture must withstand high anodic potentials under oxidizing conditions, these stress tests aim to show the corrosion resistance of AISI 442 and 446 when polarized at potentials up to 2.0 V_SHE_. Three consecutive potentiostatic polarization tests were performed at 1.5, 1.75 and 2.0 V_SHE_ for 8 h, and the corresponding chronoamperometric curves were obtained.

Following the completion of the corrosion tests, the surface of the samples and the transverse section through the chemically affected layer were examined metallographically to reveal how the microstructure of the material had degraded. The chemical composition of the two steel samples, the microstructure architecture and the topography of the corroded surfaces were analyzed by use of a TESCAN Vega 3 LM scanning electron microscope (SEM) (TESCAN Brno, s.r.o., Brno, Czech Republic) equipped with a Bruker Quantax 200 Energy Dispersive X-ray Spectroscopy (EDX) system with a Peltier-cooled XFlash 410M silicon drift detector (Bruker, Billerica, MA, USA).

As the bipolar plates from PEMWEs also experience mechanical stresses, the studied steel samples were subjected to static tensile tests using the Zwick/Roell calibrated tensile testing machine, with a capacity of 250 kN. The test temperature was 18 °C. The specimens were clamped by means of the machine’s hydraulic pans, which were located at a distance of 110 mm, with this being the reference distance of the recorded deformation. The tests were carried out at a speed of 0.1 mm/min, the force was recorded by the force cell of the machine with a measurement uncertainty of 0.1%, and the recorded deformation was taken as the displacement of the crosshead of the testing machine. For each sample, the tensile tests were repeated 3 times, and the results of the mechanical properties represent the arithmetic mean of the obtained values. The Brinell hardness values represent the arithmetic mean of 8–10 measurements on each sample. Values of the yield strength (*R*_p0.2_) have been determined according to ASTM E8/E8M-13a Standard Test Methods for Tension Testing of Metallic Materials.

## 3. Results and Discussion

### 3.1. Electrochemical Characterization

**Figure 1:**
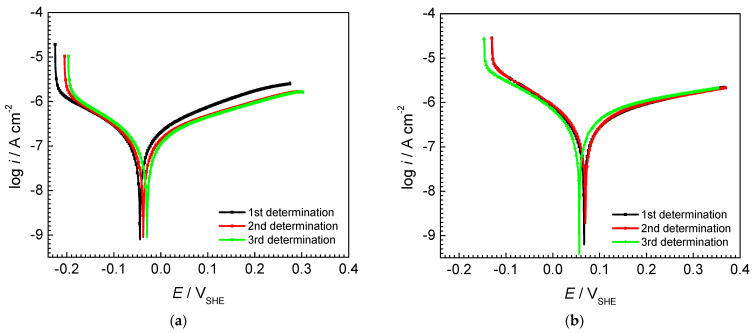

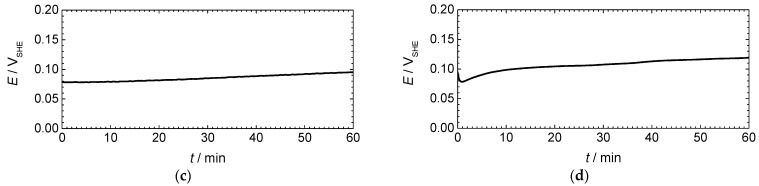
Potentiodynamic polarization curves and open circuit potential measurement in 0.1 M Na_2_SO_4_ + 0.1 ppm F^−1^ (pH = 5): (**a**,**c**) AISI 442 steel sample; (**b**,**d**) AISI 446 steel sample. Scan rate 1 mV s^–1^. Temperature 25 °C.

Figure 1 shows the potentiodynamic polarization curves and open circuit potential measurements obtained on the AISI 442 and AISI 446 ferritic stainless steel in the test solution. To verify the reproducibility of the results, three consecutive measurements were performed under the same conditions.

Based on the linear polarization curves, the following parameters that characterize the corrosion process in the given environment were calculated: corrosion current density *i_corr_*; corrosion potential *E_corr_*; cathodic and anodic Tafel slope *b_c_* and *b_a_*, respectively; and polarization resistance *R_p_*, using Equation (1). Table 2 summarizes the obtained corrosion parameters for both AISI 442 and AISI 446 for the three consecutive measurements together with their mean value and standard deviation (SD).

Corrosion current density *i_corr_* values show that AISI 442 and 446 stainless steel have good corrosion resistance in the given environment, with both meeting the target of below 1 µA cm^–2^ proposed by the US DoE [14]. The polarization resistance *Rp* is inversely proportional to the corrosion current density and implicitly to the corrosion rate of the metal alloy. According to the data presented in Table 2, the average polarization resistance recorded for the three initial tests was approximately 277 kΩ cm^2^ for the AISI 442 sample and 138 kΩ cm^2^ for the AISI 446 sample, which corresponds to the average values of the corrosion current density of 0.160 µA cm^–2^ for AISI 442 and 0.315 µA cm^–2^ for AISI 442, respectively. These results indicate better corrosion resistance of AISI 442 than AISI 446. The corrosion potential *E_corr_* characterizes the thermodynamic stability of metals or alloys in the contact environment. The more positive its value, the higher the thermodynamic stability and corrosion resistance, respectively. A higher corrosion resistance is found for AISI 446 when comparing the determined *E_corr_* values of −0.036 V for AISI 442 and 0.063 V for AISI 446. The same trend is noticed in Figure 1c,d showing the evolution over time of the open circuit potential for AISI 442 and AISI 446 steel samples. It can be seen that the OCP of both AISI 442 and AISI 446 samples slightly increases over time, stabilizing at about 0.09 V and 0.120 V, respectively. Moreover, the OCP value represents the corrosion potential in the real conditions in which the metal or alloy is found. The appearance of the oxide layer on the metal surface, due to the contact with the atmospheric oxygen, shifts the OCP towards more positive values. In the case of AISI 442 and AISI 446, the formation of a passivation oxide layer, which protects against corrosion, explains the OCP shift to more positive values. In addition, a higher OCP value of AISI 446 than AISI 442 suggests a more protective passive film. 

**Figure 2:**
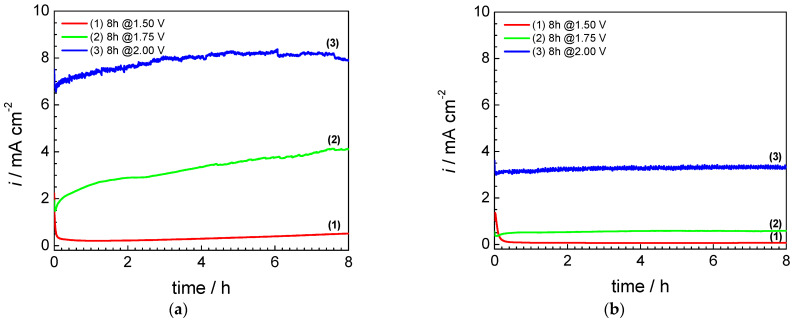
Current transients during PST at different potentials (1.5 V_SHE_, 1.75 V_SHE_ and 2.0 V_SHE_) in 0.1 M Na_2_SO_4_ + 0.1 ppm F^−1^ (pH = 5): (**a**) AISI 442 steel sample; (**b**) AISI 446 steel sample.

Ex situ potentiostatic stress tests are usually performed by applying different potential values to the BPP-solution interface for several hours to monitor the polarization resistance of the metal used as BPP [40,41,42]. The potential at which the BPP interface is polarized in an operating PEMWE can potentially reach 2 V_SHE_ [11]. When the BPP is polarized to a potential value that exceeds the reversible potential of the oxygen evolution reaction (OER), oxygen is released, which causes the local pH in the vicinity of the BPP surface to decrease. There is evidence that at near-neutral pH, such as those found in PEMWE, the local pH significantly drops near the anode during OER [43]. This causes the environment to become much more aggressive, which leads to the corrosion of stainless steels and even noble metals, such as gold [44]. A recent study of H. Becker et al. shows that the potential at the interface between the anode current collector and the BPP does not exceed 1 V during operation [17]. This would imply that the potential at which ex situ PST has to be carried out should not far exceed the reversible potential for OER, which in the solution used (pH = 5), at a temperature of 25 °C, has the value of about 0.9 V_SHE_. In fact, on BPPs, during the water electrolysis, the OER is not observed. Figure 2 shows the evolution over time of the current obtained when the AISI 442 (a) and AISI446 (b) samples were potentiostatically polarized at 1.5 V_SHE_, 1.75 V_SHE_ and 2 V_SHE_.

The results from Figure 2 indicate that when polarizing the studied stainless steel samples at 1.75 V_SHE_ and 2.0 V_SHE_, the current density through the metal-solution interface is high and increases over time for AISI 442 but shows a stable behavior for AISI 446. This is because the total measured current has a contribution from both the OER and the corrosion process [45], also supported by oxygen bubbles formed during the potentiostatic tests. The current density on AISI 442 reaches 8 mA cm^−2^ at 2.0 V_SHE_, while on AISI 446, it stabilized at a significantly lower value of 3.3 mA cm^−2^ at 2.0 V_SHE_. This behavior is caused by the higher corrosion resistance of AISI 446, as visible in the subsequent micro- and macrographs, which show the post-test SEM surface analysis of the samples evidencing the effects of the corrosion process. The situation is different at a potential value of 1.5 V_SHE_, where both samples display reduced current densities because the OER no longer takes place. In addition, a very fast decay of the current density is observed at the beginning of polarization, which is indicative of the formation of a passive, protective film [46]. For AISI 442, after the initial current decay, a slow increase is visible over time until it reaches a final value of 0.41 mA cm^−2^ after 8 h. For AISI 446, the current density stabilizes after the first 20 min and remains constant for 8 h at a much lower value of 0.06 mA cm^−2^. These observations are consistent with a more stable passive film formed on AISI 446, as compared to AISI 442, due to its higher chromium content. It has been reported that ferritic stainless steels containing a higher amount of Cr (>24%) showed the best corrosion resistance due to a very stable passivation layer, which mainly consists of chromium oxides [46,47].

**Figure 3:**
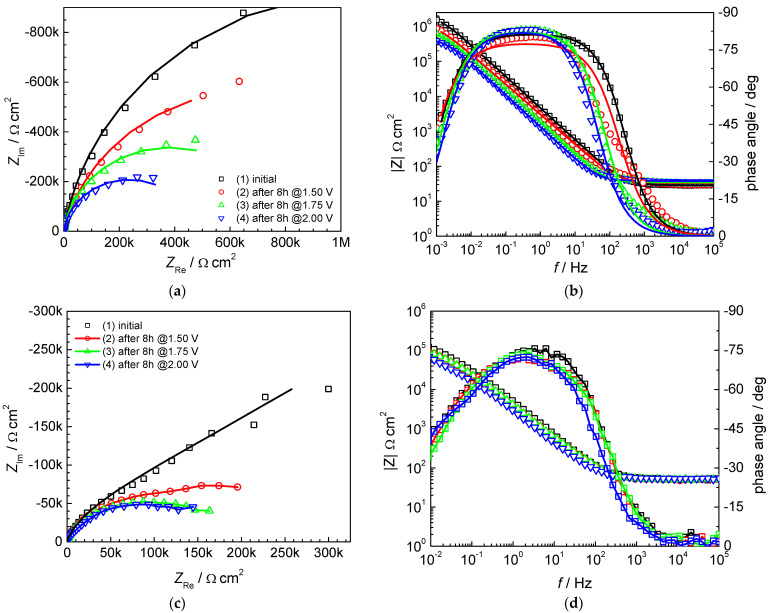
Electrochemical impedance spectra recorded at open circuit potential for the initial state and after PST in 0.1 M Na_2_SO_4_ + 0.1 ppm F^−1^ (pH = 5): (**a**) Nyquist and (**b**) Bode plots of AISI 442 steel sample; (**c**) Nyquist and (**d**) Bode plots of AISI 446 steel sample. Temperature 25 °C.

Electrochemical impedance spectroscopy measurements were conducted at open circuit potential for the initial state of AISI 442 and 446 steel samples and after the potentiostatic stress tests at different potentials. Figure 3 gives the corresponding complex plane impedance (Nyquist) plots and the absolute impedance and phase angle versus frequency (Bode) plots.

The Nyquist plots of AISI 442 and AISI 446 in Figure 3a,c point to a single capacitive semicircle with the largest diameter in the initial state and decreasing diameters after the PST. The semicircle diameter corresponds to the polarization resistance *R*_p_ and is, therefore, a direct indication of the corrosion resistance properties. The surface changes induced by corrosion during PST explain the gradual decrease in the impedance and semicircle diameter observed after the PST. The simultaneous increase in the double-layer capacitance, which is a measure of the active surface area, also supports this conclusion. A comparative analysis of Bode plots for AISI 442 (Figure 3b) and AISI 446 (Figure 3d) indicates the presence of a single time constant, implying that the mechanism underlying the corrosion process is similar in both cases. The high-frequency region of the Bode plots has the same characteristics for both samples and corresponds to the resistive response of the solution, characterized by frequency-independent values of the impedance magnitude and phase angle values near 0°. The capacitive response of the double layer dominates the lower frequency region, evidenced by an increase in the absolute impedance with a unit slope and phase angle values close to −80° for AISI 442 and to −75° for AISI 446. In order to model the impedance data, a simple electric equivalent circuit was chosen, consisting of the solution resistance *R*_S_ in series with a parallel connection of the double layer capacitance *C*_dl_ and the polarization resistance *R*_p_. Since real systems often deviate from ideal capacitive behavior, a constant phase element (CPE) replaces the double-layer capacitance to model surface inhomogeneity. The impedance of a CPE is given by *Z*_CPE_ = T^−1^(jω)^−n^, where T is a parameter related to *C*_dl_, *j* is the imaginary unit, ω is the angular frequency and *n* is an exponent between 0 and 1, describing the constant phase angle of CPE, equal to −(*n* × 90). Table 3 and Table 4 give the result of fitting for AISI 442 and AISI 446 steel samples, together with the impedance parameters errors and the goodness of fit, as expressed by the Chi-squared values. A good quality of fit between the experimental data and the selected electrical equivalent circuit is indicated by the low values of errors and of the Chi-squared parameter. The obtained values of the equivalent circuit elements point to high values of the polarization resistance, exceeding 1 MΩ cm^2^, for both AISI 442 and AISI 446 in the initial state. After performing polarization stress tests at different potentials, the polarization resistance gradually decreases because of surface modifications induced by corrosion.

### 3.2. Surface Morphology Characterization

**Figure 4:**
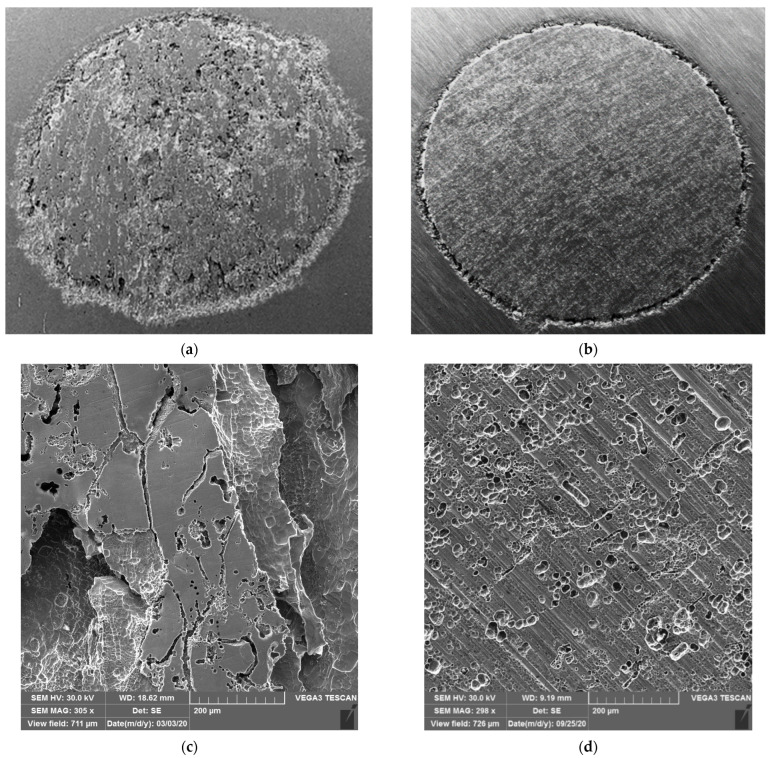
SEM topography of the surface after potentiostatic stress tests at 2 V_SHE_ for AISI 442 steel sample: (**a**) macrography, magnitude 5×; (**c**) micrography, magnitude 300×; and AISI 446 steel sample: (**b**) macrography, magnitude 5×; (**d**) micrography, magnitude 300×.

AISI 442 and AISI 446 steel samples taken from hot rolled steel sheets were subjected to quasistatic uniaxial tensile tests at room temperature, hardness tests and metallographic examinations. Table 5 shows the values of the mechanical properties such as tensile strength (*R*_m_), yield strength (*R*_p0.2_), elongation (A) and Brinell hardness (HB) for the delivery state. Figure 4 presents the SEM macro- and microstructure of AISI 442 and AISI 446 steel samples observed after the corrosion tests.

The data presented show that these steels have a higher yield strength than conventional austenitic stainless steels with 18% Cr and 8% Ni but lower ductility characteristics. Due to a carbon concentration of around 0.1% and a high chromium concentration, they are sensitive to precipitation of carbonitrides (M_23_C_6_ and Cr_2_N) and intermetallic phases (σ, χ, η) during their processing by hot working, welding and heat treatment. At the same time, for AISI 442 steel, the levels of carbon and chromium concentrations lead to the appearance of a certain proportion of martensite along with the ferritic matrix in the microstructure. Such a heterogeneous microstructure (ferrite + martensite + precipitated secondary phases) justifies the uneven arrangement of pits and corrosion microcraters on the surface of the material (Figure 4a). Since carbide precipitations are slightly larger on the boundaries of crystalline grains, the occurrence of intercrystalline corrosion is more pronounced in these regions of the material. Instead, the secondary phases precipitated inside the grains are much finer, and implicitly, the general corrosion phenomena are localized (Figure 4c).

**Figure 5:**
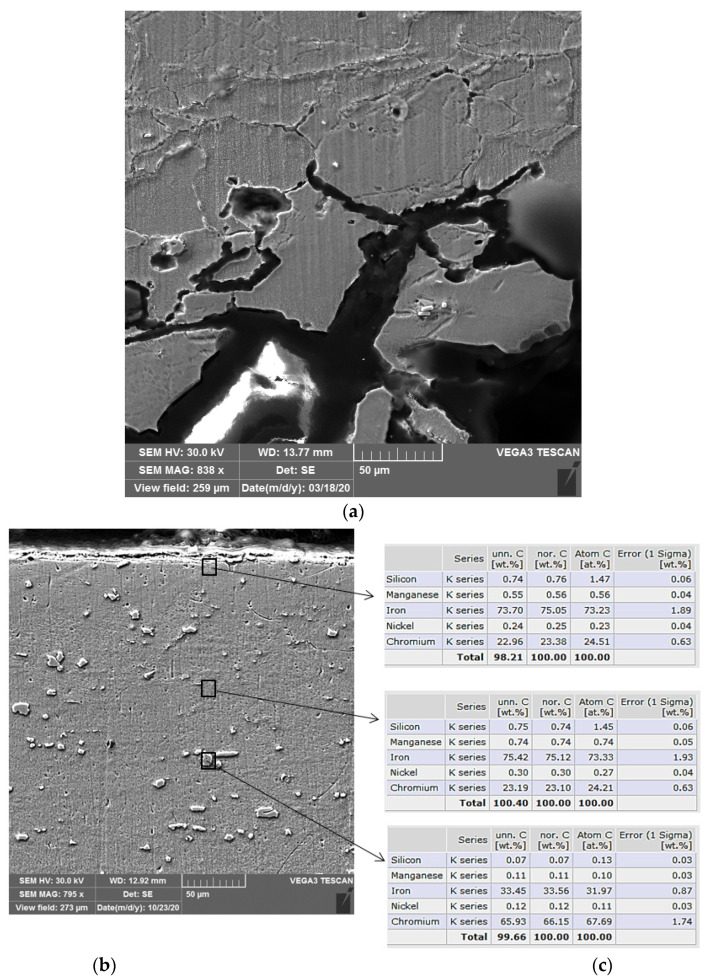
SEM microstructure of a section through the corroded (**a**) AISI 442 steel sample, (**b**) AISI 446 sample and (**c**) corresponding EDX results.

The microstructure of the AISI 442 corroded layer-base metal system shown in Figure 5a highlights the phenomenon of disaggregation of crystalline grains in the surface area of the material and the development of intercrystalline corrosion figures towards the interior areas. For AISI 446 steel, the microstructure of the system base metal corrosion-tested layer given in Figure 5b indicates excellent corrosion resistance. It is observed that it is not sensitive to either intercrystalline corrosion or pitting corrosion. In the annealed state, it has a microstructure made of ferrite and chromium carbides (Fe,Cr)_23_C_6_. Figure 5c gives the results of EDX analysis for the AISI 446 steel sample, performed in different regions as evidenced in Figure 5b: (1) surface, (2) core and (3) grain.

EDX energy dispersion analysis shows that there are no changes in the chemical composition of the surface layer compared to the core of the base material. The content in the main alloying element, which is chromium, is over 23%, the limit value for excellent corrosion resistance.

## 4. Conclusions

Electrochemical investigations revealed that the corrosion resistance of AISI 442 and 446 ferritic steels is high enough to be used as a support for BPPs in PEMWEs, as both steels displayed corrosion current densities below 1 µA cm^−2^, the target value for BPPs in PEMFC. The potentiostatic stress tests indicated better performance of AISI 446; however, the corrosion resistance at potentials higher than 1.5 V_SHE_ is deficient. To address this issue, further research on developing thin anticorrosive protective layers with low interfacial resistance is in progress. Morphology analysis showed that AISI 442 is sensitive to intercrystalline corrosion, whereas AISI 446 is more resistant to corrosion than AISI 442 due to its higher chromium content.

## Figures and Tables

**Table 1:** Elemental composition of the ferritic stainless steel samples.

Element (wt%)	C	Cr	Mn	Si	Al	P	S	N	Fe
AISI 442	0.12	22.10	0.79	0.82	0.72	0.031	0.028	-	balance
AISI 442 standard	≤0.12	17.00–19.00	≤1.00	0.70–1.40	0.70–1.20	≤0.040	<0.030	-	balance
AISI 446	0.14	24.90	1.08	0.72	-	0.032	0.026	0.18	balance
AISI 446 standard	≤0.20	23.00–27.00	≤1.50	≤1.00	-	≤0.040	≤0.030	≤0.25	balance

**Table 2:** Corrosion parameters of AISI 442 and AISI 446 steel samples obtained in 0.1 M Na_2_SO_4_ + 0.1 ppm F^−^ (pH = 5).

Corrosion Parameters	AISI 442				AISI 446			
	1	2	3	Mean (SD)	1	2	3	Mean (SD)
*i_corr_* (µA cm^−2^)	0.170	0.151	0.162	0.161 (0.009)	0.304	0.317	0.325	0.315 (0.011)
*E_corr_* (V_SHE_)	−0.045	−0.034	−0.030	−0.036 (0.008)	0.066	0.069	0.055	0.063 (0.007)
−*b_c_* (mV decade^−1^)	178	156	156	161.3 (12.7)	147	153	157	152.3 (5.0)
*b_a_* (mV decade^−1^)	232	301	311	281.3 (43.0)	289	288	287	288.0 (1.0)
*R_p_* (kΩ cm^2^)	257	295	278	276.6 (19.0)	139	137	136	137.3 (1.5)

**Table 3:** Calculated data of the circuit elements and fitting errors (between brackets) for AISI 442 steel sample in 0.1 M Na_2_SO_4_ + 0.1 ppm F^−^ (pH = 5).

Conditions	*R*_S_ (Ω cm^2^)	CPE-T (F cm^−2^ s^n−1^)	*n*	*R*_p_ (kΩ cm^2^)	*C*_dl_ (µF cm^−2^)	*Chi* ^2^
Initial	28.9 (0.3%)	4.11 × 10^−5^ (0.19%)	0.90 (0.05%)	2240 (1.1%)	20.3	8.0 × 10^−4^
after PST 8 h at 1.50 V_SHE_	32.9 (1.9%)	6.73 × 10^−5^ (1.64%)	0.86 (0.49%)	1410 (1.6%)	25.8	5.3 × 10^−2^
after PST 8 h at 1.75 V_SHE_	34.1 (1.1%)	1.04 × 10^−4^ (0.90%)	0.92 (0.28%)	760 (2.8%)	63.3	2.4 × 10^−2^
after PST 8 h at 2.00 V_SHE_	38.1 (1.0%)	1.40 × 10^−4^ (0.83%)	0.92 (0.29%)	410 (1.9%)	88.7	2.4 × 10^−2^

**Table 4:** Calculated data of the circuit elements and fitting errors (between brackets) for AISI 446 steel sample in 0.1 M Na_2_SO_4_ + 0.1 ppm F^−^ (pH = 5).

Conditions	*R*_S_ (Ω cm^2^)	CPE-T (F cm^−2^ s^n−1^)	*n*	*R*_p_ (kΩ cm^2^)	*C*_dl_ (µF cm^−2^)	*Chi* ^2^
Initial	31.1 (2.0%)	4.23 × 10^−5^ (1.61%)	0.86 (0.47%)	1470 (6.6%)	14.3	3.6 × 10^−2^
after PST 8 h at 1.50 V_SHE_	33.2 (1.7%)	6.38 × 10^−5^ (1.23%)	0.81 (0.38%)	980 (6.1%)	14.8	2.1 × 10^−2^
after PST 8 h at 1.75 V_SHE_	39.7 (1.3%)	1.97 × 10^−4^ (1.29%)	0.82 (0.51%)	775 (3.4%)	68.2	2.5 × 10^−2^
after PST 8 h at 2.00 V_SHE_	41.6 (0.7%)	3.67 × 10^−4^ (0.79%)	0.87 (0.33%)	395 (1.5%)	182.0	1.3 × 10^−2^

**Table 5:** Mechanical properties of AISI 442 and AISI 446.

Property	*R*_m_ (MPa)	*R*_p0.2_ (MPa)	A5 (%)	HB
AISI 442	564 ± 1%	345 ± 1.5%	21	194 ± 3%
AISI 446	581 ± 1%	352 ± 1.5%	19	198 ± 3%

## Data Availability

Not applicable.
